# Systematic Review of Setting-Based Interventions for Preventing Childhood Obesity

**DOI:** 10.1155/2021/4477534

**Published:** 2021-09-25

**Authors:** Khadijah Angawi, Anood Gaissi

**Affiliations:** Department of Health Services and Hospital Administration, Faculty of Economics and Administration, King Abdulaziz University, Jeddah, Saudi Arabia

## Abstract

**Introduction:**

Child obesity is recognized as one of the major public health problems globally, which demands multicomponent and comprehensive interventions. The objective of this systematic review is to evaluate, synthesize, and combine the existing evidence of various setting-based interventions across developed and developing countries that aim to prevent childhood obesity.

**Methods:**

An electronic and systematic search was conducted on setting-based interventions related to childhood obesity both in developed and developing countries. A study was considered eligible if it was a randomized controlled trial that focused on home-based, school-based, or community-based intervention for childhood obesity and published in English from 2010 to 2020. A wide range of electronic bibliographic databases, such as PubMed, Medline, Embase, and ERIC were searched. The various studies were carried out among children aged 4-18 years old. A total of 32 studies were identified; out of which 24 were school-based interventions, and the remaining were nonschool-based.

**Results:**

The studies in this review highlighted important school and nonschool-based interventions to avoid obesity among children and adolescents. School-based interventions that had considered both physical activity (PA) and diet along with home elements showed great effectiveness. These findings reveal that the specific intervention components such as nutrition education curriculum, prolonged time for PA, and upgrading self-efficacy of study participants should be considered to prevent obesity across developed and developing countries. However, the findings from nonschool-based interventions were restricted by the scarcity of the studies.

**Conclusion:**

Multisetting and multipronged strategies are required to avoid or reduce childhood obesity across the globe. However, additional studies are needed with a large sample size. Further study designs based on theory should be conducted in nonschool settings for the creation of meaningful and detailed guidelines that can support the prevention of obesity in children.

## 1. Introduction

Child obesity is the main public health problem worldwide and has affected more than 155 million children; hence, the World Health Organization (WHO) has recognized childhood obesity to be a significant challenge of the twenty-first century [1]. The incidence of childhood obesity is quite high among low- and middle-income countries undergoing nutrition and economic transition, where 20-30% of children suffer from this issue [2]. Globally, around 10% of school-going children carry additional body fat, and 25% of them are categorized as obese children [3]. Further, according to recent reports, the burden of childhood obesity has risen ten times in the last 40 years owing to changing diets and lack of exercise, which can be considered as major contributors to childhood obesity.

Childhood obesity is multifactorial [4], encompassing additive and multiplicative interactions between genes and environment that could be reflected in one's learned behaviour, food consumption, sedentary lifestyle, and sociocultural provocations [4, 5]. Such interactions that result in childhood obesity can lead to numerous outcomes such as cancer, cardiovascular diseases, hypertension, and diabetes mellitus later in life [6]. Considering the financial implications of obesity and its associated comorbidities, prevention approaches are vital, especially in developing nations that ought to manage the double jeopardy of obesity and undernutrition [2]. Therefore, governments and policymakers need to prioritize this problem by designing cost-effective and sustainable interventions [2]. However, addressing childhood obesity can be difficult due to its complex nature and multicausality, but different interventions have been tested to address childhood obesity in various studies through randomized controlled trials [2]. Besides, the existing premise also suggests that there needs to be a focus on early life stages of a child's development to break the cycle of obesity [7].

Generally, the evidence demonstrates that childhood obesity can be managed both by pharmacological (medical or surgical) and nonpharmacological interventions [8]. Nonpharmacological interventions might need to include individual, parent, family, and school-based interventions, thus making them more comprehensive and holistic [8]. In other words, the latter approach comprises alteration of behavioural factors such as improved physical activity (PA), intake of a healthy and nutritious diet, and altering environmental factors [8]. However, interventions have mostly focused on the individual level, thus ignoring the “obesogenic environment,” which is the sum of the effects that surrounding circumstances have on fostering obesity among children [9]. Altering the “obesogenic” environment could generate a more long-lasting impact on the behaviour of a child [10]. For instance, children intermingle with microenvironments including schools, homes, and neighbourhoods [9, 10]. These microlevel environments are affected by the wider macroenvironments including government policies, education and health systems, and the food industry, which are less modifiable.

Furthermore, parents play a vital role as a mediator to shape the behaviour of their children, since children spend most of the time at homes [11]. Likewise, learning settings, such as schools offer a platform to adopt a healthy lifestyle via health education and health promotion strategies throughout critical stages of child growth and development [9]. Thus, this setting-based (home, school, and community-based) interventions seem to play a crucial role to prevent or eliminate childhood obesity [9]. Despite the previous interventions that have been evaluated by many randomized controlled trials, findings of such studies are not reviewed and synthesized collectively. Therefore, it is essential to collectively assess and evaluate the effectiveness and outcomes of these interventions to give robust evidence for preventing and managing childhood obesity.

## 2. Material and Methods

The objective of this systematic review was to evaluate, synthesize, and combine the existing evidence on setting-based interventions related to childhood obesity. Guidelines specified by Preferred Reporting Items for Systematic Reviews and Meta-Analysis (PRISMA) were used to carry out this systematic review [12].

### 2.1. Inclusion and Exclusion Criteria

We carried out an electronic and systematic search in the literature review on setting-based interventions related to childhood obesity both in developed and developing countries across the world. To answer the study question, the eligibility of a study was contingent for inclusion if it was a randomized controlled trial (RCT) that was focused on nonpharmacological interventions for childhood obesity including home-based, school-based, or community-based intervention, an original research study published in English from 2010 to 2020. More specifically, we included those studies that were aimed at avoiding or controlling weight gain among children and adolescents (aged 2–19 years) in the settings such as school, preschool, community, and home by either focusing on a nutritional plan or PA or both. On the contrary, any study that had included pharmacological intervention and was published before 2007 was excluded from the review. In addition, we also excluded secondary data, letters to the editor, case reports, and grey literature from this systematic review. We grouped the eligibility criteria into four major categories using the PICOS (population, intervention, outcome, and settings) framework as given in Table 1.

### 2.2. Information Sources and Search Strategy

We started and completed a systematic search of published articles in 2020. A wide range of electronic bibliographic databases such as PubMed, Medline, Embase, and ERIC was searched. We also explored references of pertinent reviews along with the database search. The primary outcome of the analysis was a reduction in childhood obesity that was mainly assessed by the prevalence of obesity at the end line. This was evaluated by the change in factors including body mass index (BMI), waist circumference, body fat percentage, and skinfold thickness from starting to the end of the study. We also grouped into four major categories of the same PICOS (population, intervention, outcome, and settings) framework. We prepiloted the search strategies without any restrictions by year of publication, geographic area or country, or other sociodemographic characteristics.

We labeled a study with favorable or positive findings if all outcomes of particular interest in the intervention arm demonstrated a statistically significant decline in adiposity. On the other hand, we classified a study with mixed results if more than one of the outcomes such as BMI, waist circumference, and skinfold thickness in the intervention group revealed a positive effect. The study was labeled to have negative findings when all the outcomes in the intervention group upsurged significantly, and lastly, we considered a study with no effect on the outcome when there were no significant differences found between the intervention and controlled group for the particular outcome.

We identified a combination of Medical Subject Heading (MeSH) keywords and text words. These were also clustered into four major groups based on the categories of population, intervention, outcome, and settings. The most prevalent search key terms found in abstracts and titles comprised of “setting based interventions for childhood obesity,” “childhood obesity AND school-based intervention,” “childhood obesity AND home-based intervention,” “childhood obesity AND community-based intervention,” “intervention for childhood obesity,” and “sustainable interventions for childhood obesity.” Further, we consulted with a librarian to generate a search in four different parts. The first part was restricted to search terms specific to the primary outcome such as “overweight/obese”; the second part was for the terms limited to the population of the study including “children” and “pediatric”; the third part was related to the terminology relevant for the intervention such as “prevention” and “control”; and the last term was related to the setting including “Preschool” or “home-based.”

Besides, we also considered using diverse wordings of main concepts such as childhood obesity vs. obesity among children to obtain pertinent research papers. This was followed by combining these major concepts using combinations (AND, OR) relevant to the research question. Moreover, to detect more research articles, we also used truncation (∗) with the same root word. While executing the search strategy, a filter was applied to retrieve articles in English language only. Additionally, restrictions were applied on publication period, age group, and type of studies to include eligible studies in our systematic review.

### 2.3. Data Abstraction

We imported all appropriate research studies into the reference manager software (Endnote™) file. Titles were screened for duplicates in this software. We did not consider the abstracts for further review, which did not explicitly explore the study objective. Finally, we obtained and examined the full-text articles of the remaining relevant articles. This was followed by abstracting and summarizing the articles that met the eligibility criteria using a standardized proforma. Thus, after the process of removing duplicates, title, and abstract screening, we removed papers that were beyond the scope of this review as guided by inclusion criteria. Besides, the bibliography of the remaining studies was also checked and scrutinized to evade missing any useful studies. This process of searching the articles was carried out independently by the reviewers, and their judgments and extracted summaries were matched to identify the differences and resolve these accordingly.

Independent reviewers filled a standardized data extraction sheet for eligible research articles. The reviewers compared the data extraction tables to ensure including the imperative findings of the eligible studies and pilot tested the data extraction sheet before starting the process of data extraction. Besides, prevailing research articles on the chosen topic were reviewed to describe objects of the data extraction proforma. Any discrepancies between the two reviewers were resolved by discussion and agreement. The abstracted data comprised the author, reference, publication year, and title; total sample size; sample size by gender if applicable; medical field, a method to measure outcome; factors of satisfaction and factors of dissatisfaction; and ranking of the included medical faculty members.

## 3. Results

### 3.1. Findings of the Search Strategy

**Figure 1:**
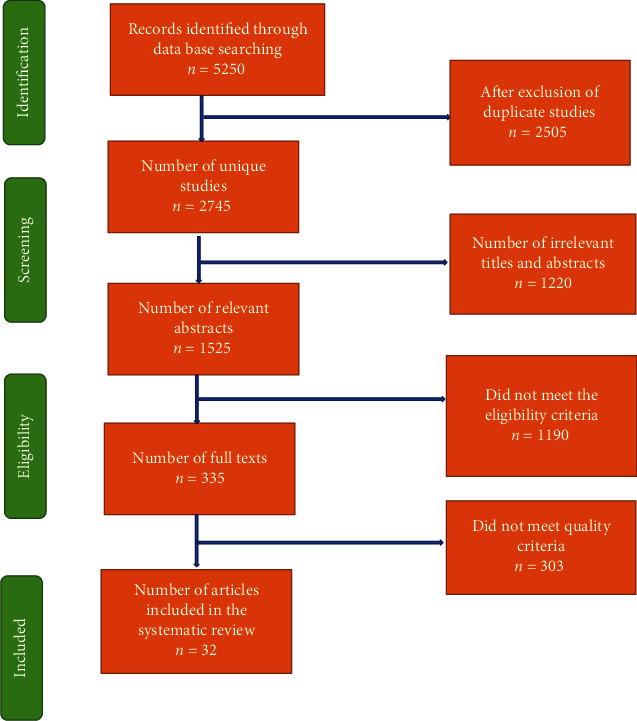
Flow chart summarizing the identification and selection of randomized controlled trials for systematic review.

We screened the identified articles initially by titles and then by abstracts, and finally, we carried out full-text article assessment. Articles that did not meet the eligibility criteria were not included. As a result, our initial search identified 5,250 citations in different databases; however, 2,505 articles were duplicates that were removed. Of the remaining 2,745 unique studies, we reviewed titles and abstracts and found 1,525 relevant abstracts. Upon reviewing abstracts, 1,190 articles did not meet the eligibility criteria while reviewing the abstracts, and 303 did not meet eligibility after reviewing full texts. Hence, we were able to retrieve full texts for 32 articles, which were included in the review as shown in Figure 1.

### 3.2. Characteristics of the Eligible Studies

With respect to the setting, of these thirty-two studies, twenty-four were undertaken in the school settings, and eight were done in nonschool setting, of which five were done in the preschool setting, 1 was conducted in the community, and only two were carried out in the home. Regarding the effect of the intervention on the outcome, seven studies revealed a favourable effect on the outcome with positive findings, twelve studies had combined results with mixed effects on the outcomes, and thirteen studies did not find any difference between the intervention and controlled arm. Positive results mean the given intervention resulted in the reduction of all obesity-related outcomes such as BMI, skinfold thickness or waist circumference, percentage of body fat, and prevalence of obesity in the intervention group. On the other hand, negative effects mean that the reduction in these outcomes was noticed in the control group rather than the intervention group. Lastly, mixed-effects mean that not all obesity-related outcomes were improved in the intervention group; rather, it implies that at least one of these outcomes improved. Details of the type of intervention, length of intervention, length of follow-up, age groups of children involved in the intervention, outcome of the intervention, and effect of the intervention on the outcome are presented as shown in Table 2.

### 3.3. Findings of the School-Based Interventions

Overall, 24 RCTs were evaluated interventions to control weight gain in school settings [13–36]. Generally, the results of the school-based RCTs were diverse with most studies revealing positive or combined findings [14, 15, 17–23, 25–30, 33], and several studies demonstrated no difference between intervention and control group. Almost all RCTs that reported statistically significant findings used both PA and dietary interventions [18, 19, 21, 32]. More specifically, the intervention components included an education curriculum for nutrition, a prolonged time for PA [18, 19, 21], and upgrading self-efficacy of study participants [18, 20]. On the contrary, almost 50% of RCTs conducted in school-based settings with no significant findings utilized mixed interventions of PA and diet [16, 32, 35, 36] and the rest of the studies focused entirely on diet, PA, or chasing body measurement [13, 31, 34]. These studies showing insignificant findings also emphasized supplementary approaches implemented in the respective RCTs such as changes at the policy level social marketing, and communication about the health status of study participants [16, 34–36]. Most RCTs with combined findings used a combination of PA and interventions [13–15, 17, 22, 25, 27, 30] such as education curriculum [14, 15, 17, 22–25, 27–30] or prolonged PA time [15, 22, 24–29, 33], and environmental changes [14, 22].

In numerous RCTs with insignificant findings, the interventions were offered for short period and the follow-up time ranged from 5 weeks to 7 years [16, 32–35]. With respect to the outcome, the studies with statistically significant findings reported only BMI or its *z*-score as a unique primary outcome. In contrast, the studies with insignificant results reported a range of outcomes such as waist circumference [13, 17, 25, 26, 28, 30], skinfold thickness [33], body fat percentage [13, 23, 24, 26], and incidence of overweight or obesity in addition to the BMI [14, 22, 23, 26, 28, 30, 32, 34, 35]. Most of the RCTs with insignificant findings considered children as well as adolescents and were conducted in countries such as China, Argentina, Australia, Spain, and Denmark [13, 16, 31, 34, 35]. Around 50% of the RCTs showed combined findings, and intervention effect was based on factors such as duration of follow-up [17, 24, 33], type of intervention [14, 15], and the outcome of interest [17, 22, 24–30] or inclusion of extraneous factors in the final model [23].

### 3.4. Findings for the Nonschool-Based Interventions

#### 3.4.1. Childcare and Preschool-Based Interventions

Overall, five RCTs tested the proposed interventions to manage weight gain in preschool settings [37–41], and findings of the RCTs had a wide range of positive [37–39] and insignificant findings [40, 41]. The duration of follow-up varied between 9 months [37, 38] to 2 years [39]. The majority of RCTs in childcare settings documented favourable findings that emphasized PA [37, 38] entirely except for one study that advocated a multipronged approach by including environmental interventions at the policy level at childcare settings [39]. The intervention focused on both healthy diet and PA policies. These policies promoted a healthy drink intake among school staff and children, focusing on water as the main beverage and limiting juice intake to once per week. The intervention also focused on a daily intake of fresh fruits and/or vegetables, encouraged PA for >60 min/d, and reduced screen time limited to <30 min/week [39]. This study noted a significant increase in the nutritional consumption of fruit and vegetable of obese children in the intervention group compared to the control group [39]. These second RCTs that had favourable findings were carried out in USA, considered BMI as the main outcome, and included children from low-income families. The interventions included cognitive-behaviourally based PA that was conducted among 4-5-year-old children. Age-appropriate cognitive-behavioural techniques related to PA were tested in these RCTs and were carried out in the USA. The interventions were associated with a significantly greater percentage of moderate-to-vigorous and vigorous PA in a preschool day. The intervention was also associated with a significant reduction in BMI, with effect sizes greatest in overweight and obese children [37]. In contrast, the RCTs with no difference between intervention and control group (*n* = 2) delivered a two-week education program focusing on a nutritious diet and PA [40, 41]. However, one study also added family-centred intervention by arranging classes for parents on a nutritious diet and PA [40].

#### 3.4.2. Community and Home-Based Interventions

Several community and home-based RCTs were carried out in the USA by trained staff and involved families with children through partnering with different community settings. One RCT was conducted in the community setting in the USA where public recreation centres were randomly allocated to a health-promoting intervention that focused on exercise-centre policies, PA programs, and services while incorporating family-centred approaches including visits at home and group workshops [42]. The BMI, diet, and PA for each child were monitored from baselines to 2-year postbaseline. After following the participants, there were no significant difference found in the outcome between the two groups (*P* = 0.13) among children aged 5 to 8 years.

Two home-based RCTs conducted in the USA [43, 44] evaluated interventions to manage weight gain, both of which did not find any difference between the intervention and treatment groups, although both RCTs were executed in community centres and was directed towards families. For instance, one RCT involved 160 families with children aged 8-12 years that were randomly allocated to an intervention that focused on their nutritional education and meal planning and encouraged to reduce screen time while having meals [43]. The researchers did not find any significant difference in the outcome between intervention and control group after 12 months (*P* = 0.43) or at the end of 21 months (*P* = 0.21).

## 4. Discussion

We reviewed 32 RCTs with diverse interventions that were tested around the world in this systematic review. The initiatives were intended to reduce or control weight gain in children and teenagers, with the most of the RCTs (*n* = 24) predominantly carried out in the school setting. The majority of the studies (*n* = 17) found statistically significant and favourable results of the respective interventions for at least one obesity-related outcome among school-based studies. These results reveal that schools should be considered as focal points for interventions to prevent childhood obesity [45, 46]. Students devote 50% of their time and eat at least one-third of their everyday calories at school, and current facilities can be used by educational institutions to prevent obesity without major alterations to the timetable or lifestyle of the child [46].

A blended diet and PA approach were implemented by most of the school-based RCTs, which showed a positive or mixed result. This is analogous to previous evidence that indicates that a mixed approach to diet and PA may be more effective compared to a single strategy [47]. With an emphasis on the strength of action, the benefit of integrated approaches over single methods should be further discussed. An additional home setting was also included in most school-based RCTs in which the results of the intervention were found to be beneficial; this finding is in concordance with existing evidence that acknowledges the role of the family members and home setting in affecting the children and adolescents' health behaviour [48, 49].

One possible reason that several studies did not seem to be able to identify alterations in the outcomes related to obesity may partly explain studies with combined findings. In this systematic review, over half of the studies did not have a well-defined primary outcome. Among the RCTs with combined findings for which a primary outcome was established, 9 RCTs found that the intervention had a statistically significant positive effect. Results from RCTs conducted in nonschool environments have been less coherent. This is because there have been somewhat fewer programs, completed in nonschool settings, with six studies in preschool settings. In general, in both PA-only and mixed diet and PA approaches, the best preschool trials showed modest proof of efficacy. All the preschool-based studies were carried out in low-income nations that focused on minority groups. While there were only two RCTs performed solely in the home, most school-based studies with favourable outcomes reported for intervention in a secondary home environment. The value of the home setting should not be overlooked based on the minimal data available. Future studies are needed to test the interventions addressing different contexts (e.g., school and home) to help control childhood obesity.

Regarding the duration of treatments, it should be noted that some studies included intervention components that have altered the policy, environment, or personnel education, which may have had repercussions that have continued beyond completion of assessments. Such interventions might continue beyond the study period, and it is worth assessing the sustainability of such programs. This is because, apart from the short-term or medium-term efficacy of the RCTs, the viability of initiatives must be addressed. In addition, helping children sustain healthy habits after the study is over to avoid gain in weight is important. Furthermore, policy and environmental changes that eliminate barriers to modifying individual behaviours will help change the obesogenic circumstances that lead to increased weight [50].

### 4.1. Strengths and Limitations

Our review has unique strengths. First, we included RCTs to have meaningful and valid estimates for the outcomes. Second, we carried out a comprehensive review of obesity-related intervention by incorporating both school and nonschool settings. Third, we included studies from both developing and developed countries to assess the efficacy of interventions that have been implemented across diverse settings. However, the study results need to be interpreted considering few limitations. First, the included RCTs had variation in the study parameters such as length of follow-up, type of outcome, and several study participants that rendered difficult for a researcher to compare the findings across various studies. Second, we limited our review to studies that were published in the English language, and studies published in a non-English language might have different findings than we noticed, thereby introducing publication bias.

## 5. Conclusion

The findings of the review indicate reasonable evidence to endorse school-based approaches that incorporate components of both diet and PA and as well as a home environment to minimize childhood obesity. However, further research is required with strong study designs that are based on theory and carried out in nonschool settings for the creation of meaningful and detailed guidelines that can support the prevention of obesity in children. Multisetting and multipronged strategies are required for the most positive outcomes to avoid or reduce childhood obesity across the globe. Since most of the evidence with favourable findings was coming from developed countries, therefore, specific attention to implementing future studies in developing countries is warranted. This systematic review has important policy implications to reduce obesity among children both in developed and developing countries. The environment in all settings such as schools, preschools, and communities need to be conducive in a way that should not encourage children to adapt obesogenic environment. This means that schools in developed countries should have meal plans with nutritious diets and a physical environment for doing physical activity. Activities such as sports should be encouraged in schools with enough time scheduled for different types of physical activities. A school curriculum should be adapted which students are taught about the importance of being active and consuming a healthy diet. These cost-effective interventions are also applicable in the developing countries; however, such countries might need more cost-effective interventions such as physical activity in the form of walking for 30 minutes each day. Additionally, developing countries also need to integrate some modules of improving physical activity and avoiding unhealthy diets in their existing curriculum. More macrolevel actions are required at the policy level to avoid selling unhealthy foods to children and adolescents. Moreover, governments should focus on building local parks and playgrounds in neighbourhoods to keep children physically active and engaged.

## Figures and Tables

**Table 1:** Characteristics of the RCTs included in the systematic review (*n* = 32).

Study	Year	Country	*n*	Setting
Nemet et al.	2013	Israel	342	School-based
Bonsergent et al.	2013	France	5354	School-based
Cunha et al.	2013	Brazil	478	School-based
Dewar et al.	2013	Australia	357	School-based
Fairclough et al.	2013	England	318	School-based
Meng et al.	2013	China	9750	School-based
Safdie et al.	2013	Mexico	886	School-based
Rappaport et al.	2013	USA	8504	School-based
Rausch Herscovici et al.	2013	Argentina	405	School-based
Annesi et al.	2013	USA	1154	Preschool based
Annesi et al.	2013	USA	273	Preschool based
Fitzgibbon et al.	2013	USA	146	Preschool based
Kain et al. 2014	2014	Chile	1474	School-based
Martínez-Vizcaíno et al.	2014	Spain	1070	School-based
Santos et al.	2014	Canada	687	School-based
Simon et al.	2014	France	954	School-based
Tarro et al.	2014	Spain	2350	School-based
Llaurado et al.	2014	Spain	916	School-based
Lubnas et al.	2014	Australia	361	School-based
Meyer et al.	2014	Switzerland	502	School-based
Xu et al.	2014	China	1182	School-based
Elder et al.	2014	USA	541	Community-based
Cao et al.	2015	China	1854	School-based
Greve et al.	2015	Denmark	18423	School-based
Fulkerson et al.	2015	USA	160	Home/community
Llargués et al.	2016	Spain	566	School-based
Leme et al.	2016	Brazil	253	School-based
Bogart et al.	2016	USA	2439	School-based
Natale et al.	2016	USA	1211	Preschool-based
Kong et al.	2016	USA	618	Preschool-based
Hull et al.	2016	USA	272	Home/community
Annesi et al.	2017	USA	141	School-based

**Table 2:** The key features of included RCTs and main findings of the studies (*n* = 32).

Study	Age (years)/grade	Intervention type	Intervention duration	Length of follow-up	Outcome	Effect on outcome
Nemet et al.	5 years	PA and diet	One year	One and two years	BMI and percentile of BMI	Positive results
Bonsergent et al.	10th to 11th grades	PA and diet	Two years	Two years	BMI *Z* score, BMI, obesity, or overweight	Mixed results
Cunha et al.	5^th^ grades	Dietary intervention only	Nine months	Nine months	BMI, body fat percentage, and obesity	Mixed results
Dewar et al.	12 to 14 years	PA and diet	One year	One year	BMI, body fat percentage, and *z* score of BMI	Mixed results
Fairclough et al.	10 to 11 years	PA and diet	Twenty weeks	Thirty weeks	BMI, *Z* score for BMI, and waist circumference	Mixed results
Meng et al.	Six to thirteen years	PA and diet	One year	One year	BMI, *Z* score for BMI, obesity, or overweight	Mixed results
Safdie et al.	4 to 5 years	PA and diet	Eighteen months	Eighteen months	BMI, obesity, or overweight	Mixed results
Rappaport et al.	5 to 12 years	Dietary intervention only	Seventeen months	Twenty-nine months	*Z* score for BMI and obesity, or overweight	No difference between two groups
Rausch Herscovici et al.	9 to 11 years	PA and diet	Six months	Six months	*Z* score for BMI and BMI	No difference between two groups
Annesi et al.	4 to 5 years	PA	Nine months	Nine months	BMI	Positive results
Annesi et al.	4 to 5 years	PA	Nine months	Nine months	BMI	Positive results
Fitzgibbon et al.	3 to 5 years	PA and diet	Fourteen weeks	One year	BMI and *Z* score of BMI	No difference between two groups
Kain et al.	6 to 8 years	PA and diet	One year	One year	BMI *Z* score	Positive results
Martínez-Vizcaíno et al.	Eight to ten years	PA	Nine months	Nine months	BMI, obesity, body fat percentage, and waist circumference	Mixed results
Santos et al.	6 to 12 years	PA and diet	Ten months	Ten months	*Z* score of BMI, waist circumference	Mixed results
Simon et al.	Six grades	PA only	Four years	Six and half years	*Z* score for BMI and waist circumference	Mixed results
Tarro et al.	2nd grade	PA and diet	Twenty-eight months	Twenty-eight months	BMI, *Z* score for BMI obesity or overweight, and waist circumference	Mixed results
Llaurado et al.	Seven to eight years	PA and diet	Twenty-two months	Twenty-two months	*Z* score for BMI and obesity or overweight	No difference between two groups
Lubnas et al.	12 to 14 years	PA	Twenty weeks	8 months	BMI, *Z* score for BMI and percentage of body weight	No difference between two groups
Meyer et al.	One grade	PA	Nine months	Nine months and three years	BMI, skinfold thickness, and waist circumference	No difference between two groups
Xu et al.	Four school grades	PA and diet	Ten months	Ten months	BMI and obesity or overweight	No difference between two groups
Elder et al.	5 to 8 years	PA and diet	Two years	Two years	BMI, *Z* score for BMI, waist circumference, and percentage of body weight	No difference between two groups
Cao et al.	One grade	PA and diet	Three years	Three years	BMI *Z* score, BMI, obesity, or overweight	Mixed results
Greve et al.	5 to 17 years	Other	Three years	Two years	BMI, obesity, or overweight	No difference between two groups
Fulkerson et al.	8 to 12 years	PA and diet	One year	One year and twenty-one months	*Z* score for BMI	No difference between two groups
Llargués et al.	5 to 6 years	PA and diet	Two years	Six years	BMI	Positive results
Leme et al.	14 to 18 years	PA and diet	Six months	Six months	BMI, *Z* score for BMI, and waist circumference	Mixed results
Bogart et al.	Grade seven	PA and diet	Five weeks	Two years	Percentile of BMI	No difference between two groups
Natale et al.	2 to 5 years	PA and diet	Two years	Two years	Percentile of BMI	Positive results
Kong et al.	3 to 5 years	PA and diet	Fourteen weeks	Sixteen months	BMI and *Z* score of BMI	No difference between two groups
Hull et al.	5 to 7 years	PA and diet	One year	Six and sixteen months	*Z* score for BMI and BMI	No difference between two groups
Annesi et al.	9 to 12 years	PA and diet	Nine months	Nine months	BMI	Positive results

## Data Availability

The data supporting this systematic review are from previously reported studies and datasets, which have been cited within the manuscript. The processed data are freely available online.
